# Evaluation of Durability of Transparent Graphene Electrodes Fabricated on Different Flexible Substrates for Chronic in vivo Experiments

**DOI:** 10.1109/TBME.2020.2979475

**Published:** 2020-03-17

**Authors:** David Ding, Yichen Lu, Ruoyu Zhao, Xin Liu, Chawina De-Eknamkul, Chi Ren, Armaghan Mehrsa, Takaki Komiyama, Duygu Kuzum

**Affiliations:** Department of Electrical and Computer Engineering, University of California San Diego, La Jolla, CA 92093 USA; Department of Electrical and Computer Engineering, University of California San Diego, La Jolla, CA 92093 USA; Department of Electrical and Computer Engineering, University of California San Diego, La Jolla, CA 92093 USA; Department of Electrical and Computer Engineering, University of California San Diego, La Jolla, CA 92093 USA; Nanoengineering Department, University of California San Diego, La Jolla, CA 92093 USA.; Neurobiology Section, University of California San Diego, La Jolla, CA 92093 USA; Department of Electrical and Computer Engineering, University of California San Diego, La Jolla, CA 92093 USA; Neurobiology Section, University of California San Diego, La Jolla, CA 92093 USA; Department of Electrical and Computer Engineering, University of California San Diego, La Jolla, CA 92093 USA

**Keywords:** graphene, accelerated aging, microelectrode arrays, chronic reliability, polyethylene terephthalate (PET), SU-8, neural interface

## Abstract

**Objective:**

To investigate chronic durability of transparent graphene electrodes fabricated on polyethylene terephthalate (PET) and SU-8 substrates for chronic *in vivo* studies.

**Methods:**

We perform systematic accelerated aging tests to understand the chronic reliability and failure modes of transparent graphene microelectrode arrays built on PET and SU-8 substrates. We employ graphene microelectrodes fabricated on PET substrate in chronic *in vivo* experiments with transgenic mice.

**Results:**

Our results show that graphene microelectrodes fabricated on PET substrate work reliably after 30 days accelerated aging test performed at 87 °C, equivalent to 960 days *in vivo* lifetime. We demonstrate stable chronic recordings of cortical potentials in multimodal imaging/recording experiments using transparent graphene microelectrodes fabricated on PET substrate. On the other hand, graphene microelectrode arrays built on SU-8 substrate exhibit extensive crack formation across microelectrode sites and wires after one to two weeks, resulting in total failure of recording capability for chronic studies.

**Conclusion:**

PET shows superior reliability as a substrate for graphene microelectrode arrays for chronic *in vivo* experiments.

**Significance:**

Graphene is a unique neural interface material enabling cross-talk free integration of electrical and optical recording and stimulation techniques in the same experiment. To date, graphene-based microelectrode arrays have been demonstrated in various multi-modal acute experiments involving electrophysiological sensing or stimulation, optical imaging and optogenetics stimulation. Understanding chronic reliability of graphene-based transparent interfaces is very important to expand the use of this technology for long-term behavioral studies with animal models.

## I. Introduction

Electrophysiology has been one of the major tools for basic neuroscience research and clinical practice for decades. There have been vast technological advances in electrode technologies, allowing for high-temporal resolution recording of neuronal spikes and local field potentials generated by neuronal ensembles. Optical techniques such as multi-photon imaging has opened new avenues for high-spatial resolution mapping of neural activity from hundreds of neurons [1, 2]. Combining electrical recordings with optical methods such as multi-photon microscopy or optogenetics is crucial to study neural dynamics at multiple spatial and temporal scales. To that end, graphene-based optically transparent microelectrode arrays built on flexible polymer substrates have been developed and employed in various acute *in vivo* experiments [3–8]. Graphene is an optically transparent conductor, offering several outstanding properties, such as flexibility [9], high conductivity [10] and biocompatibility [11, 12], making it attractive for neural interface applications. Optically transparent graphene microelectrode arrays have been shown to enable simultaneous multi-modal imaging and electrical recording with high spatiotemporal resolution in acute studies. However, further studies investigating the chronic reliability of graphene-based transparent neural interfaces is critical for adaptation of this technology to long-term behavioral studies with animal models [13].

Transparent graphene arrays can be fabricated on different types of flexible polymer substrates, which exhibit optical transparency in wavelength ranges from 450 nm to 850 nm, commonly used in optical imaging and stimulation experiments. Different types of polymers including polyimide [4, 14], SU-8 [15], PET [5, 6], Parylene-C [16] and polydimethylsiloxane (PDMS) [17] have been investigated for fabrication of metal-based flexible microelectrode arrays in the past. Among those, polyimide is not preferred due to its yellow color and absorption peak around 450 nm, overlapping with wavelength of interest for optical imaging and stimulation [4]. Parylene-C has been shown to work in chronic settings. However, the dry etch processes needed to pattern Parylene-C also etches graphene, leading to complex fabrication processes involving sacrificial layers to protect graphene. Stretchable PDMS substrates are widely used for wearable sensors but softness and stretchability of PDMS increases the risk of cracks forming on monolayer graphene. Therefore, in this study we focus on the remaining two polymer substrates PET and SU-8, which meet the requirements for transparency, fabrication compatibility and mechanical support to the monolayer graphene electrodes. We fabricate transparent graphene microelectrode arrays on PET and SU-8 substrates using similar process steps to perform systematic studies to understand the chronic reliability and failure modes of transparent graphene microelectrode arrays built on PET and SU-8 substrates. In order to investigate the chronic reliability of these arrays comparatively, we employ an accelerated aging test, with an aging factor of 32 times. We examine various modes of failure including delamination, cracking, leakage using systematic optical microscope and scanning electron microscopy (SEM) inspection and electrochemical impedance spectroscopy tests performed over 30 days, 2.63 years of equivalent aging. Finally, we perform *in vivo* chronic animal experiments on mice with simultaneous 2-photon imaging and electrical recordings using the PET graphene electrode array and evaluate the stability of the electrode impedance and the recording noise over time.

## II. Methods

### A. Fabrication

**Figure 1.**
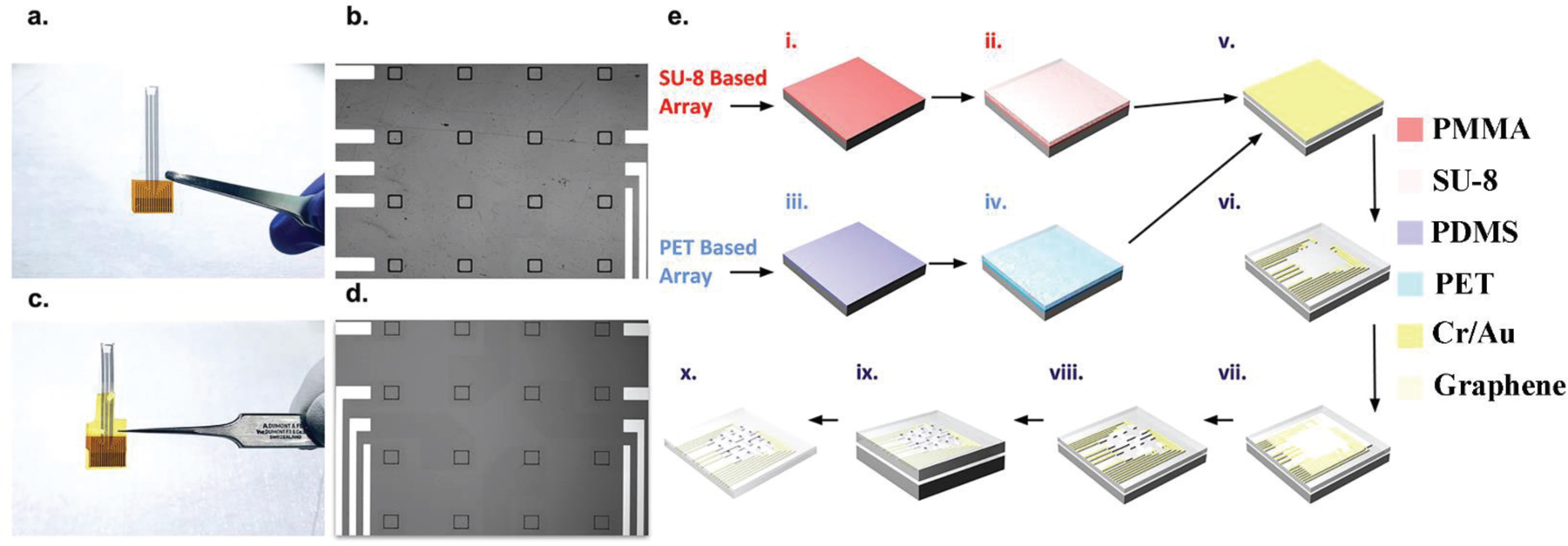
Macro and micro images of graphene microelectrodes fabricated on SU-8 and PET substrates, along with the fabrication flow. (a,b) Macro and micro image of graphene microelectrode array fabricated on PET substrate. Each array contains 16 electrode openings, each with a size of 100 µm x 100 µm. (c,d) Macro and micro image of graphene microelectrode array fabricated on SU-8 substrate. (e) i) PMGI sacrificial layer spin-coated onto a silicon wafer for graphene microelectrode array fabricated on SU-8 substrate. ii) SU-8 substrate spin-coated onto the wafer. iii) PDMS sacrificial layer spin-coated onto a silicon wafer for graphene microelectrode array fabricated on PET substrate. iv) PET substrate mechanically applied onto the wafer. v) 10 nm chromium and 100 nm gold deposited for both arrays. vi) Metal wires patterned using photolithography and excess metal etched. vii) Monolayer CVD graphene transferred onto the wafer using the ‘‘bubbling’’ method. viii) Graphene pattemed using AZ1512/PMGI bilayer lithography and etched with oxygen plasma etching. ix) SU-8 encapsulation spin-coated and openings patterned using photolithography. x) Sacrificial layer lift-off for final array.

We fabricated 16-channel graphene microelectrode arrays with a 100 μm electrode size and 400 μm spacing on SU-8 and PET substrates (Figures 1a–d). Besides the substrate fabrication, the arrays go through the same fabrication flow. For the graphene microelectrode array with an SU-8 substrate, a sacrificial layer is created by spin-coating polymethylglutarimide (PMGI) resists on a silicon wafer. A 10 μm thick SU-8 layer is spin-coated and defined with photolithography on the sacrificial layer to create the substrate. The substrate is baked incrementally from 125 °C to 135 °C in order to bond the SU-8 [18]. For the graphene microelectrode array with a PET substrate, PDMS is spin-coated on a silicon wafer as the adhesive layer. A 50 μm thick PET substrate layer is then mechanically applied onto the wafer. For both microelectrode arrays, 10 nm chromium and 100 nm gold are deposited onto the substrate and patterned into metal wires and contact pads using photolithography and wet-etching processes. Chemical vapor deposited (CVD) monolayer graphene is transferred onto the wafer using the ‘‘bubbling’’ transfer method [5, 19], and the graphene pads are patterned with AZ1512/PMGI bilayer lithography and oxygen plasma etching. A ‘‘four-step’’ cleaning process’’ is then deployed with the wafers going through 3 solvent bathes of AZ 1-Methyl-2-pyrrolidone (AZ NMP), Remover PG, acetone, and finally a 10-cycle isopropanol/deionized water rinse. A second 10 μm thick SU-8 encapsulation layer is then spin-coated, and the electrode openings/contact pads are defined using photolithography as well. The encapsulation layer is also hard-baked at 150 °C to bond the second layer of SU-8. The graphene microelectrode array fabricated on a PET substrate is mechanically lifted-off the wafer, while the graphene microelectrode array fabricated on an SU-8 substrate is immersed in AZ NMP remover for its lift-off process. A detailed schematic of both graphene array’s fabrication is shown in Figure 1e.

### B. Characterization and comparisons

**Figure 2.**
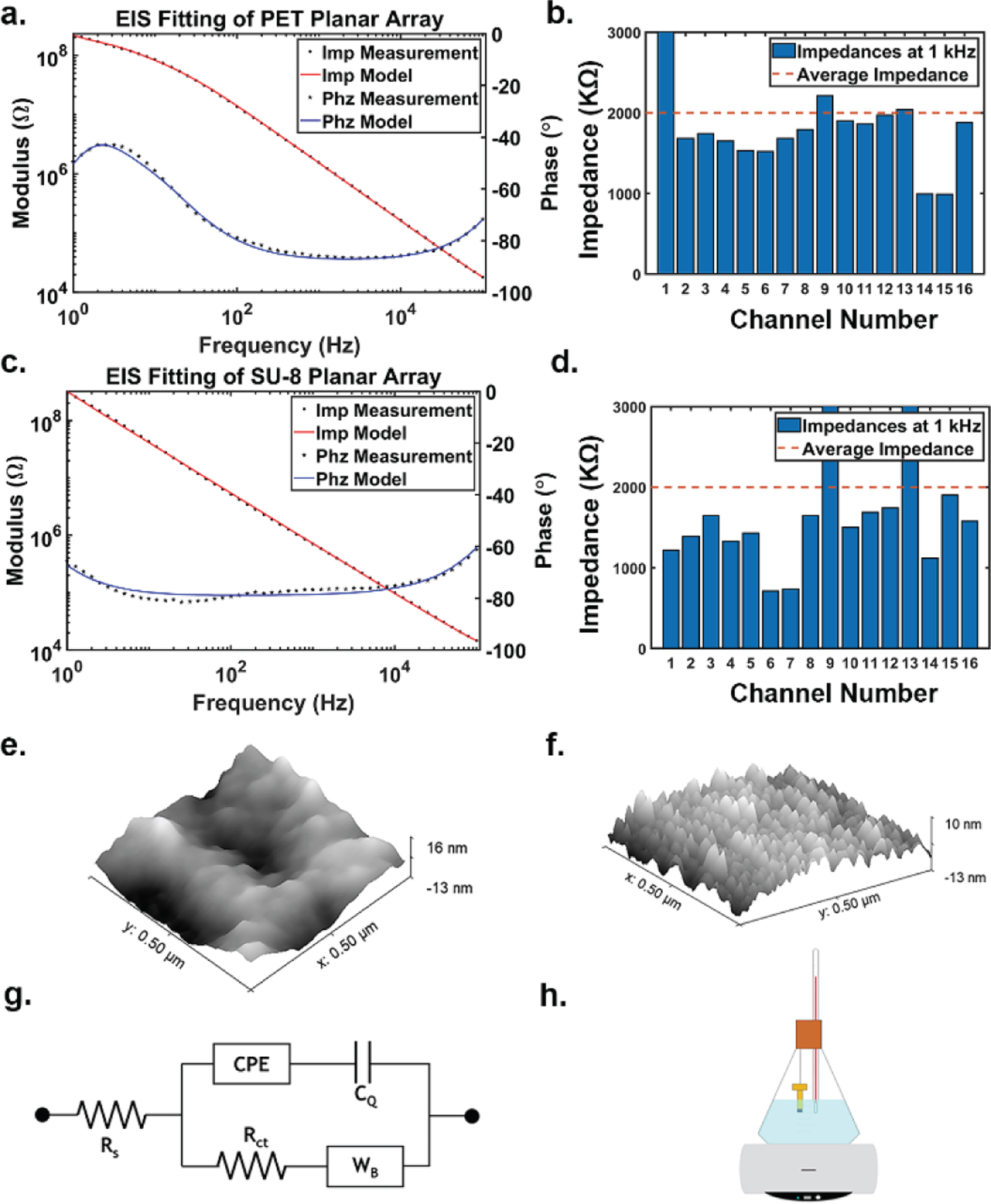
Electrochemical and surface characterization of both graphene microelectrode arrays and equivalent circuit model. (a) EIS results from 1 Hz to 100 kHz of graphene microelectrode array fabricated on PET substrate along with equivalent model fitting. (b) 1 kHz impedance bar chart for sample graphene microelectrode array on PET substrate. (c) EIS results from 1 Hz to 100 kHz of graphene microelectrode array fabricated on SU-8 substrate along with equivalent model fitting. (d) 1 kHz impedance bar chart for sample graphene microelectrode array on SU-8 substrate. (e) Tapping-mode atomic force microscopy (AFM) 3-dimensional photograph of PET substrate. (f) AFM 3D image of SU-8 substrate. (g) Equivalent circuit model for graphene microelectrode arrays, regardless of substrate. R_s_ is the series resistance. CPE is the constant phase element representing the Helmholtz double-layer capacitance. C_q_ represents quantum capacitance. R_ct_ represents the charge-transfer resistance, and Wb is a bounded Warburg element representing any diffusion processes. (h) Accelerated aging test schematic showing the graphene microelectrode arrays suspended in 87 °C PBS solution. A hotplate is used to keep the PBS constantly heated, and a thermometer is held next to the arrays in order to confirm the temperature. The beaker is enclosed by a silicone stopper in order to prevent temperature changes and evaporation.

Both graphene microelectrodes fabricated on a PET substrate and SU-8 substrate were characterized using electrochemical impedance spectroscopy (EIS) in 0.01 M phosphate buffered saline (PBS) to study the graphene/electrolyte interface (Figures 2a–d). Impedance results for microelectrodes built on PET and SU-8 substrates from 1 Hz to 100 kHz are shown in Fig. 2a and Fig. 2c. Impedance histograms at 1 kHz are shown in Fig. 2b and Fig. 2d, respectively. To maintain a good signal-to-noise ratio (SNR) for electrical recordings, an electrode’s impedance at 1 kHz should be below 2 MΩ when using Intan acquisition systems [5]. The results suggest that graphene microelectrodes show low impedance and high yield (~90% working channels) regardless of the substrate used. We investigated whether use of different substrates changes the effective surface area for the graphene microelectrodes. Atomic force microscopy (AFM) scanning results for PET and SU-8 substrates are shown in Figure 2e and Figure 2f, respectively. Although PET and SU-8 show different surface morphology, the effective surface areas calculated based on AFM results have shown similar values, with SU-8 being slightly larger (7% increase compared to geometrical surface area) than PET (1.5% increase compared to geometrical surface area). We incorporated these effective surface area results in the equivalent circuit model (Figure 2g). In the equivalent circuit model fitting, R_s_ mimics the series resistance of the solution, the constant phase element (CPE) represents the Helmholtz double-layer capacitance (C_dl_), the bounded Warburg element (W_b_) simulates any diffusion processes occurring, and R_ct_ is the charge-transfer resistance, which simulates any faradaic reactions [20]. Representative parameter values for both graphene microelectrode arrays fabricated on a PET substrate and SU-8 substrate are listed in Table I.

Graphene gives rise to an additional quantum capacitance component represented by C_q_ [6, 21] in series with C_dl_. Computational values of C_dl_ (around 10 μF cm^−2^) are larger than computational values of C_q_ (around 1–4 μF cm^−2^) [21, 22]. C_dl_ is experimentally measured using Au electrodes [23], and C_q_ is computed by fitting EIS results to the equivalent circuit model shown in Fig. 2g. The quantum capacitance dominates the impedance in both graphene microelectrode arrays, regardless of the substrate. Our experimental values are thus consistent with theoretical predictions. The R_ct_ and W_b_ values are both large, so the electrode behaves capacitively, which is consistent with well-known characteristics of graphene.

### C. Accelerated Aging Test

Accelerated aging tests have been used to simulate *in vivo* lifetime for implantable devices [24–27]. The general theory states that holding a device in elevated temperatures exponentially increases the rate of chemical reactions, thus speeding up the degradation of the materials to simulate long-term defects within a short experimental time. A commonly accepted approach is to use Eq. (1) as the factor of increased aging [24]. ΔT is equal to the difference between the temperature the device is held at (T) and the reference temperature (T_ref_). For our study, T_ref_ is set at 37 °C, for that is the average body temperature of mice.

(1) $$f=2^{\Delta T/10}$$

To simulate *in vivo* environments, we suspended the microelectrode arrays in a glass beaker and immersed them in 0.01 M PBS at pH 7.4, a buffer solution commonly used in accelerated aging tests due to the constant pH level [26]. The set up was enclosed by a silicone stopper due to its heat resistance [28], and the beaker was placed on a hot plate (Figure 2h). The solution was kept at 87 °C for an accelerated aging factor of 32 according to Eq. (1), so that 1 month in the aging test is equivalent to 32 months *in vivo*. Four graphene microelectrode arrays fabricated on a PET substrate and four graphene microelectrode arrays fabricated on an SU-8 substrate were used for a 30-day accelerated aging test. The impedance of working channels was tracked every three days for PET and every day for the SU-8 arrays which showed early failure.

### D. *Graphene Electrode Implantation for Chronic* in vivo *Experiments*

All procedures were in accordance with protocols approved by the UCSD Institutional Animal Care and Use Committee and the guidelines of the National Institutes of Health. Adult mice (cross between CaMKIIa-tTA (JAX 003010) and tetO-GCaMP6s (JAX 024742), 2 months old) were anesthetized with isoflurance (3% for induction and 1% for maintenance) and a circular piece of scalp was removed. A custom-built head-post was implanted to the exposed skull (1 mm posterior to lambda) with cyanoacrylate glue and cemented with dental acrylic (Lang Dental). A stainless-steel screw (F000CE156, J.I. Morris) was implanted over right olfactory bulb as reference. A square craniotomy (4 × 4 mm^2^) was made over the left hemisphere and the graphene microelectrode array was implanted to cover visual cortices. A glass imaging window was placed on top of the electrode array and further secured with vetbond (3 M) and dental acrylic. To protect the array while the mice were in the home cage, a custom 3D-printed case was employed. Electrophysiological data were recorded by the RHD2000 amplifier board and RHD2000 evaluation system (Intan Technologies), which has a large input impedance which allows us to achieve high SNR with our relatively high impedance electrodes. It is important to note that relatively high impedance of graphene electrodes necessitates recording systems with large input impedance to prevent signal attenuation. The data was processed using custom Matlab code.

## III. Results

### A. Microelectrode Arrays on PET

**Figure 3.**
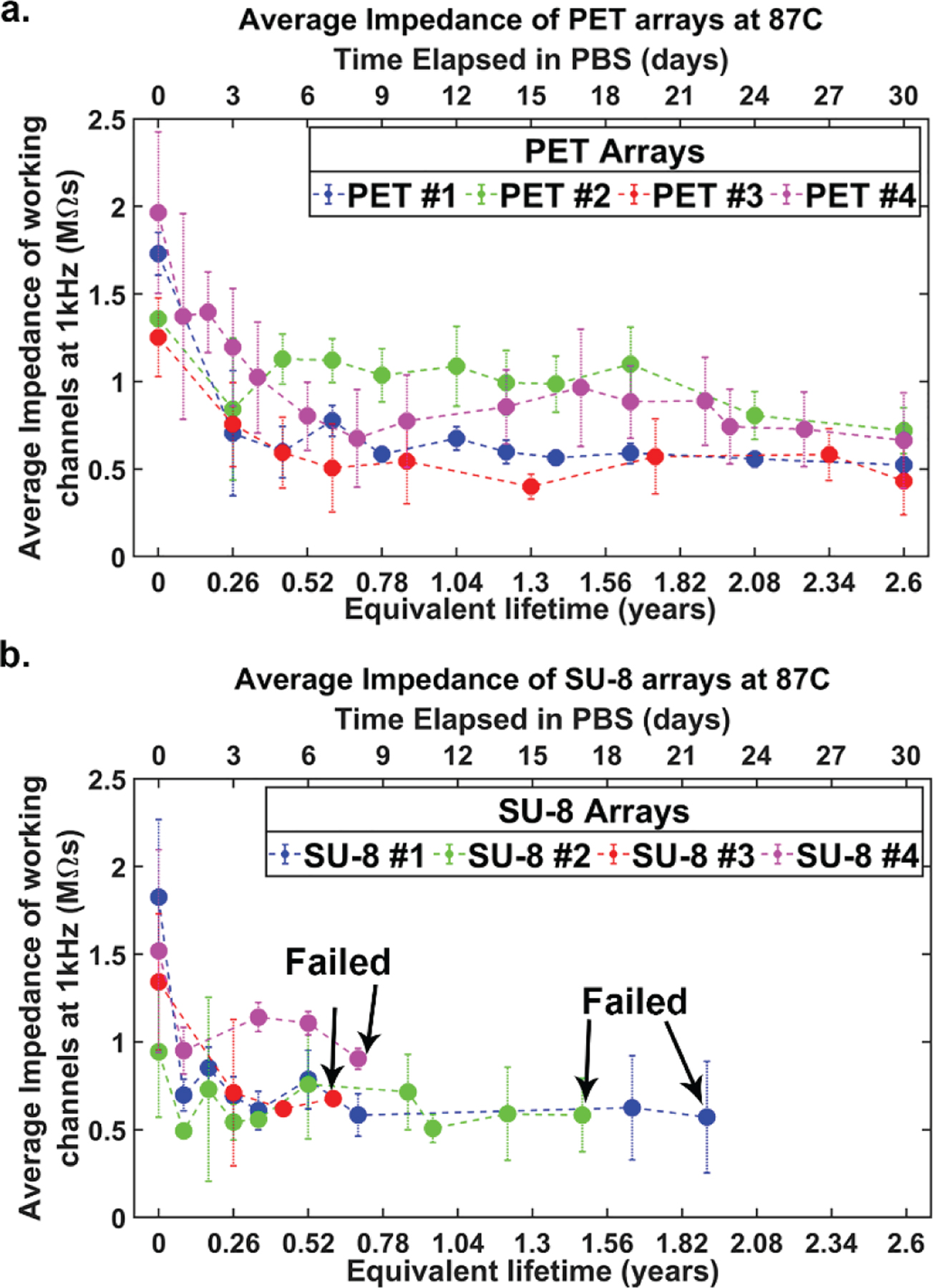
Impedance graphs over accelerated aging test. (a) Average impedances along with the standard deviation of four graphene microelectrode arrays fabricated on PET substrate over 30 days of 2.63 equivalent years *in vivo*. (b) Average impedances along with the standard deviation of four graphene microelectrode arrays fabricated on SU-8 substrate over 30 days of 2.63 equivalent years *in vivo*. Failed arrows indicate when each array failed during the aging test. None of the four arrays made it the full 30 days.

**Figure 4.**
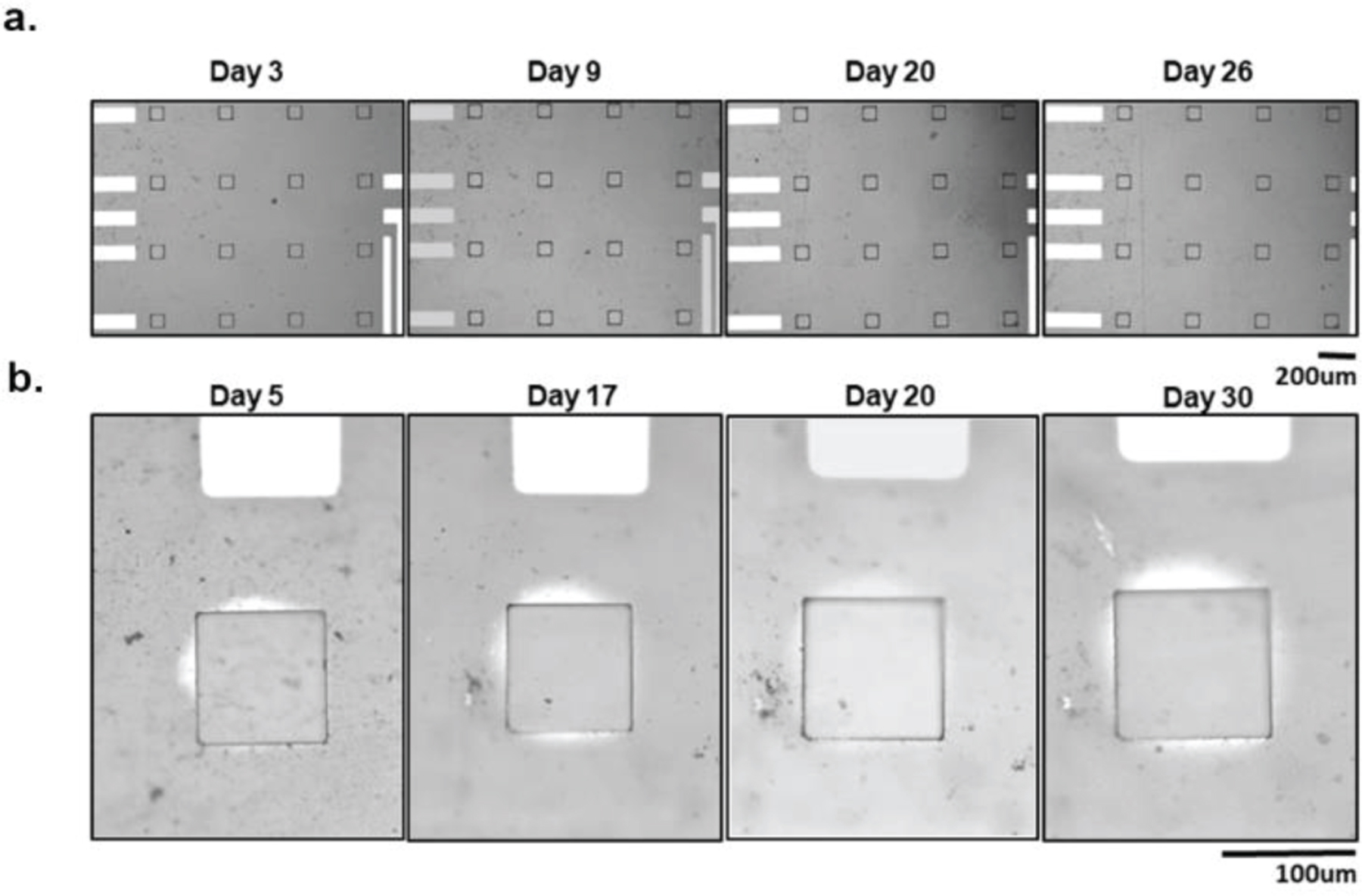
Graphene microelectrode arrays fabricated on PET substrate micro images over time. (a) One graphene microelectrode array fabricated on PET substrate micro images over 30 days in the accelerated aging test. No visual differences between start and end of the experiment. (b) One graphene microelectrode array fabricated on PET substrate that displayed signs of PBS permeation. One electrode on the array is tracked over the course of 30 days. The PBS permeation gets slightly larger overtime but is never able to reach other electrode sites or the wires.

All the microelectrode arrays fabricated on a PET substrate were working after the 30-day accelerated aging test. The average impedance of the microelectrodes over the 30-days is plotted along with the standard deviation in Figure 3a. The average impedance across all the channels from the four different arrays after the full duration of the test is 585 kΩ. The average impedance of the microelectrode arrays fabricated on PET substrate showed an initial drop but stabilized after a few days. This initial impedance drop is a common observation for electrodes soaked in solution for longer durations [29, 30]. The graphene arrays fabricated on PET substrate successfully completed the aging study with stable impedance characteristics, reaching an equivalent lifetime over 960 days (2.63 years). Figure 4a shows microscope images of a representative PET arrays captured on different days during one-month long study. Graphene microelectrodes fabricated on PET substrate did not exhibit any delamination, cracking or other forms of degradation until the end of the aging test.

However, one of the PET arrays (array number 3) exhibited visual PBS permeation between the PET substrate and SU-8 encapsulation layer (Figure 4b). Liquid intrusion and substrate delamination are common sources of delamination for thin-film interfaces [31–33]. The delamination can be attributed to the weaker adhesion between the SU-8 encapsulation layer and PET substrate, leading to the PBS leakage beneath the encapsulation surface. In order to precisely measure permeation distance, we processed microscope images in grey scale and adjusted the threshold to clearly identify the saline permeation as seen in Supplementary Figure S1. The size of the permeated region increased over the 30 days for the representative channel, with the maximum permeation distance reaching around 18 μm and percentage area increased to a maximum of ~14%. Since that is much smaller than interelectrode spacing (400 μm) between electrode sites, this minor permeation does not pose the risk of electrical shorting between the channels for our design. The impedance of all the PET arrays is stable at the end of the test, with an average impedance of 585 kΩ. The array which showed permeations has a lower average impedance (432 kΩ) consistent with the slight increase in the effective electrode area [34]. In summary, the results of the accelerated aging tests show that monolayer graphene microelectrodes fabricated on PET substrates demonstrate long-term durability required for chronic *in vivo* experiments.

### B. Microelectrode Arrays on SU-8

**Figure 5.**
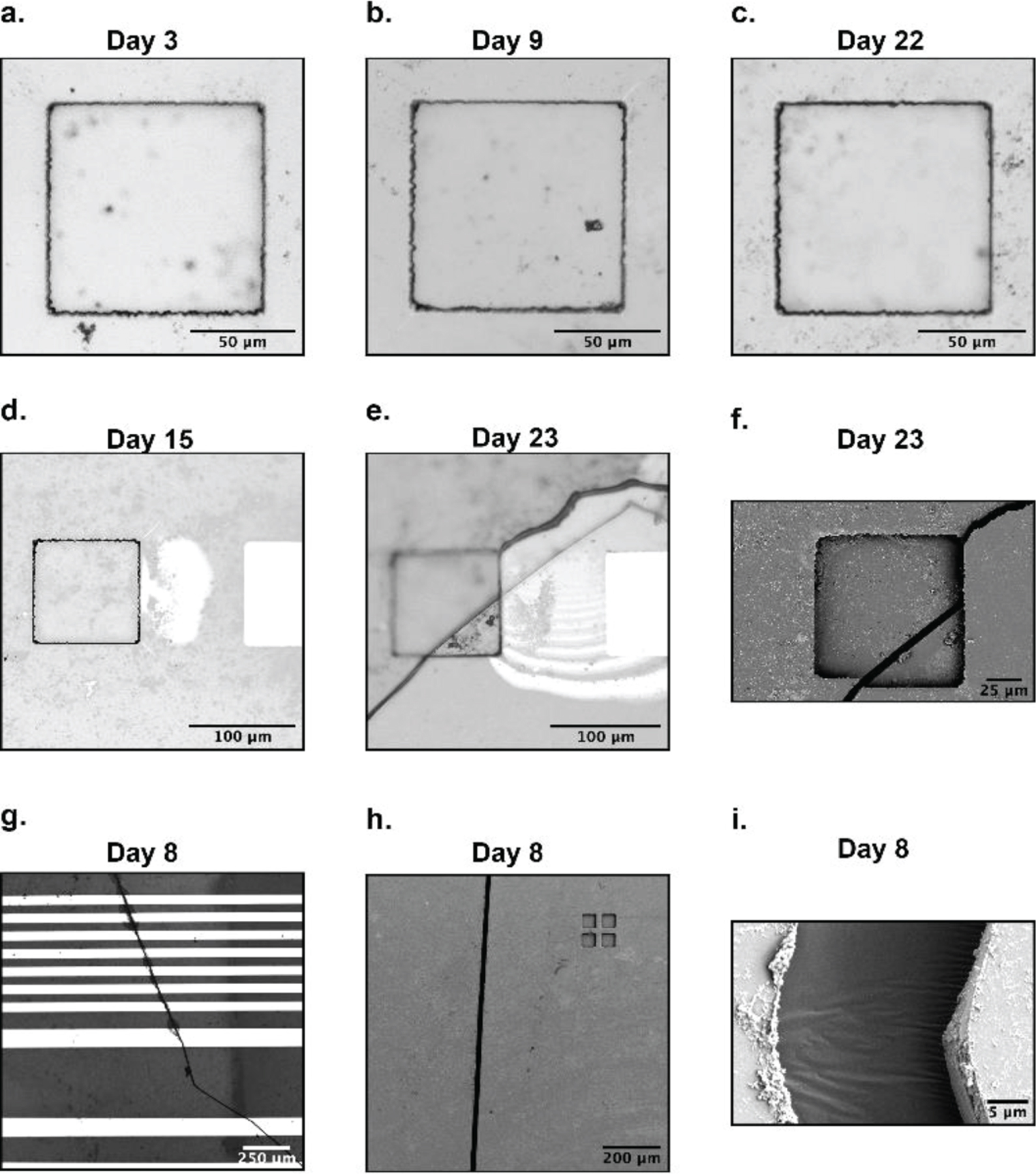
Graphene microelectrode arrays fabricated on SU-8 substrate post accelerated aging results. (a,b,c) A single electrode site on a graphene microelectrode array fabricated on SU-8 substrate over 22 days. (d,e,f) A separate electrode site on the same array in (a,b,c) tracked from day 15 to day 23. A large crack spontaneously formed on day 23, causing leakage and device failure. An SEM image of the crack is shown in (f), where a noticeable vertical distortion is created on the electrode site. (g,h,i) A different graphene microelectrode array fabricated on SU-8 substrate that suffered from crack formation along its metal wires. (g) shows the cracks cleanly penetrating numerous wires. SEM images in (h,i) clearly defines the crack’s thickness and depth.

All four graphene microelectrode arrays fabricated on SU-8 substrate succumbed to crack failures prior to the conclusion of the 30-day accelerated aging test. The average impedance of SU-8 arrays over the 30-days is plotted along with the standard deviation in Figure 3b. Graphene electrodes fabricated on SU-8 substrate also showed an initial drop like the PET arrays but stabilized after a few days. The final average impedance of all working channels across the arrays is 684 kΩ. None of the arrays showed any signs of PBS delamination (Figures 5a–c). However, two forms of crack formations occurred: cracks over the electrode site (Figures 5d–f) and cracks over the gold/chromium wires (Figures 5g–i). The two arrays with cracks over the electrode site had a longer equivalent aging time (1.93 and 1.49 years), while the arrays with cracks over the wires failed earlier on (0.70 and 0.61 years). On the one hand, Figure 5f shows SEM images of an example crack over the electrode site. The crack completely deforms and penetrates the electrode site. On the other hand, Figure 5i shows that the cracks over the wires are deep and wide (around 20 μm). The crack fully punctures through the SU-8 encapsulation layer as well as the gold/chromium wires. This causes open failure and prevents any recordings of electrical signals or impedance measurements, which renders the arrays nonworking.

A summary of all the arrays detailing equivalent lifetime age and failure mechanisms is listed in Table II. Our study concludes that graphene arrays fabricated on PET substrate meets the lifetime and durability requirements for chronic studies in animal models. In contrast, graphene arrays fabricated on SU-8 substrate consistently failed due to crack formation, making them unsuitable for chronic animal experiments.

Some studies in literature have suggested SU-8 as a reliable material for neural applications [15, 35, 36] but crack formation in SU-8 substrates have also been reported in many studies [37–39]. In our study, both graphene microelectrode arrays have an SU-8 encapsulation layer, but only the graphene microelectrode arrays fabricated on SU-8 substrate are prone to crack formation. Mechanical properties of several soft substrates are listed in Supplementary Table I. The two factors which requires attention are the thermal expansion coefficients and the fracture strain (elongation at break) percentages [40]. PET and SU-8 have similar thermal expansion coefficients, but there is a significant distinction between PET and SU-8’s fracture strain (elongation at break percentages). While PET and SU-8 behave thermo-mechanically similarly, PET is mechanically much more compliant. Our experimental observation that the cracks only form on the top encapsulation layer suggests that the SU-8/PET combination exhibits better thermomechanical stability compared to the SU8 encapsulation/substrate configuration. It has also been reported in a separate study that a PET substrate is able to form an adaptive layer when SU-8 is fabricated on top due to its similar thermal expansion coefficient as well as less adhesion between the two layers [41], compared to an SU-8 substrate with SU-8 encapsulation layer. The 7% difference in surface roughness between PET and SU-8 also has effects on the adhesion strength and crack formation. The SU-8’s larger effective surface area leads to a stronger adhesion strength [42–44], which correlates to our observation that only PET showed a minor delamination issue. On the other hand, it has been shown that surface roughness does not contribute to crack formation failure modes [45–47]. The risk of cracking for a graphene microelectrode array fabricated on SU-8 is notably high (100% in our study) such that the device does not demonstrate chronic reliability.

### C. Chronic In Vivo Experiments

**Figure 6:**
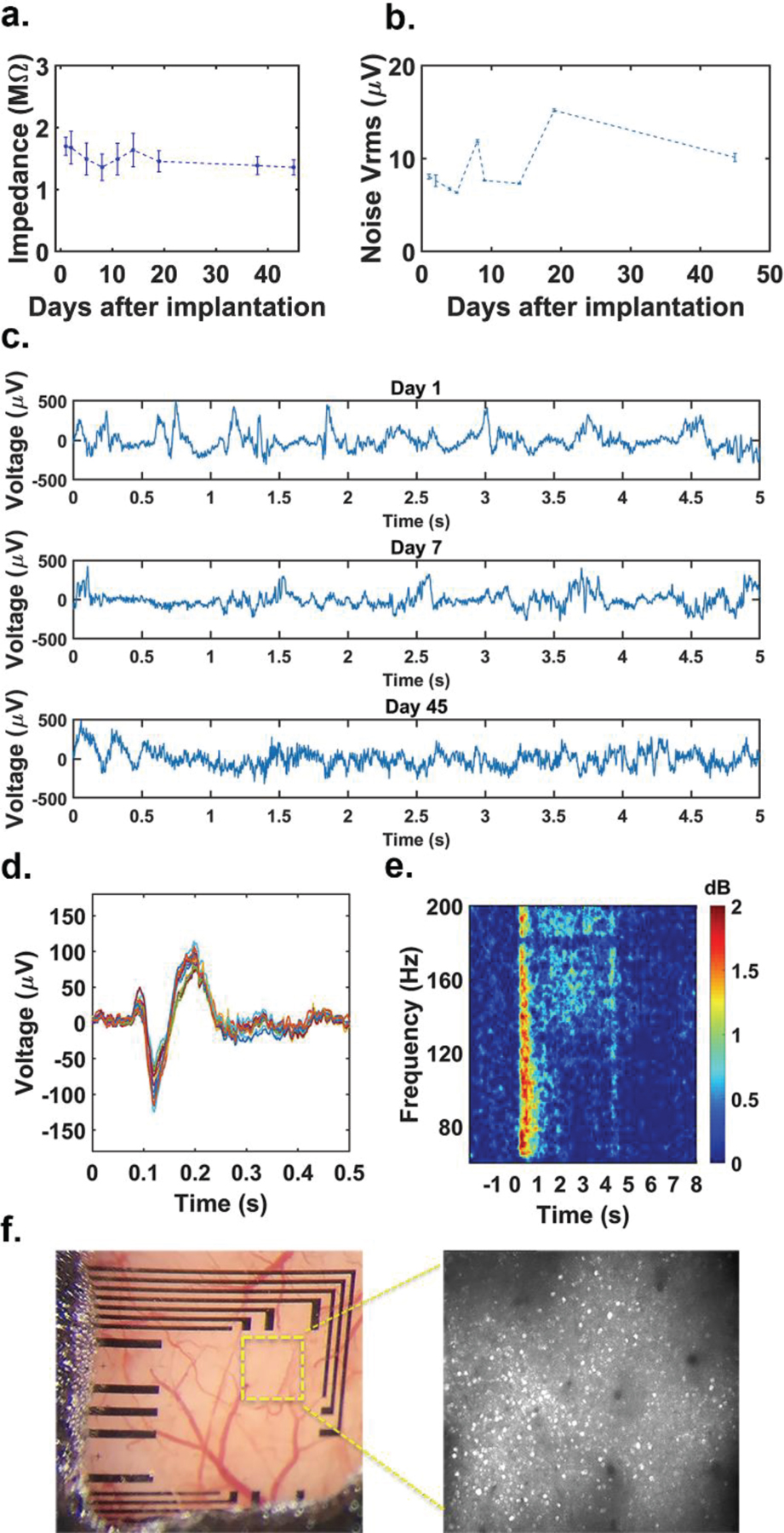
Chronic in vivo implantation of graphene microelectrode array fabricated on PET substrate. (a) Average impedance of working channels of transparent graphene microelectrode arrays built on PET over the course of 45 days after implantation. (b) Root-mean-square (rms) values of the noise of the electrical recordings throughout the course of the *in vivo* experiment. (c) Representative electrocorticography (ECoG) recordings on days 1, 7, and 45. All recordings were taken while the animal was awake. (d) ECoG visual responses under awake state. Stimulus-induced trial-averaged ECoG response of the awake mice. There is a typical on-response ~70 ms after the given stimulus at 0 seconds. (e) Stimulus-induced trial-averaged frequency response spectrogram. A persistent power increase between [140–200 Hz] is observed. (f) Pictures of the imaging window for chronic 2-photon imaging taken 13 days after implantation. Image on the right shows 2-photon microscope image taken at 400 µm depth under the yellow outlined area.

Based on the results of the accelerated aging tests, transparent graphene microelectrode arrays fabricated on a PET substrate were determined to be more reliable for chronic *in vivo* studies. Therefore, for chronic *in vivo* experiments, we only used transparent graphene microelectrodes fabricated on PET substrates. To further evaluate the reliability of the transparent graphene microelectrode arrays in chronic imaging and recording experiments, we implanted the graphene microelectrodes over the visual cortex of transgenic mice. The transgenic mice express GCaMP6s [48], which is a green fluorescent calcium biosensor that is widely used to image neural activity, in the cortical neurons. We have previously shown that graphene microelectrode arrays do not generate light-induced artifacts under 2-photon imaging or optical stimulation [6]. The photo-induced currents are intrinsically very weak and fast in graphene enabling artifact-free integration of 2-photon imaging or optogenetics with electrical recordings [49]. Similar to the results obtained during accelerated aging tests, the impedance of the electrodes drops in the first few days after the initial implantation and stays stable throughout the course of 45 days (Figure 6a). On the other hand, the noise of the array rises slightly after the implantation and remains stable towards the later days (Figure 6b). With the low noise, we successfully recorded the spontaneous electrocorticography (ECoG) signals with high fidelity for 45 days after implantation (Figure 6c, Supplementary Figure S2).

In addition, we observed stimulus-induced responses in multiple ECoG channels, showing delayed on-responses and elevated signal power across wide frequency bands (Figures 6d, 6e). During the experiment, we gave visual stimulus to the mice. Square-wave drifting grating stimuli (100% contrast, 0.04 cycles/degree, 3 cycles/sec, covering entire contralateral receptive field) were presented on a LCD monitor (30 × 38 cm^2^) positioned 15 cm away from the right eye using Psychtoolbox [50]. One of 12 orientations (30° apart) was presented for 4 seconds on each trial in pseudorandom order. Inter-stimulus-interval was varied between 7–9 seconds. We presented each orientation at least 10 times in a session. We also performed simultaneous high quality 2-photon calcium imaging in Layer IV of the visual cortex at 400 μm depth without causing light-induced artifacts in recordings. The imaging frame rate is set to ~29 Hz, and the imaging area covers 786 × 846 μm^2^. The neuron cell bodies are clearly identified due to the high transparency of the graphene electrodes and PET substrate (Figure 6f). Our results demonstrate that transparent graphene microelectrodes on PET achieve stable impedance, low noise and artifact-free recordings in chronic *in vivo* experiments combining electrophysiological recordings with 2-photon microscopy.

## IV. Conclusion

We performed systematic accelerated aging tests to investigate reliability of transparent graphene microelectrode arrays fabricated on PET and SU-8 substrates. Graphene microelectrodes fabricated on PET substrate show high reliability with all functioning channels after 30 days of accelerated aging or 2.63 years equivalent *in vivo* lifetime. On the other hand, graphene microelectrodes fabricated on SU-8 substrate exhibited failures due to different forms of crack formation across the electrodes or wires. We further demonstrated the reliability of graphene microelectrode arrays fabricated on PET in chronic 45-day *in vivo* studies in mice. After 45 days, graphene electrodes still exhibit low noise and impedance and can reliably record ECoG during calcium imaging. Our studies conclude that compared to other substrate materials, PET shows superior reliability and is therefore an ideal substrate of the graphene microelectrodes arrays for chronic *in vivo* studies, combining simultaneous electrophysiology and optical imaging.

## Supplementary Material

supp1-2979475

## Figures and Tables

**TABLE I:** EQUIVALENT CIRCUIT MODEL FITTING VALUES FOR GRAPHENE MICROELECTRODE ARRAYS

Substrate	R_s_ [KΩ]	C_dl_ [µF cm^−2^]	A	W [MΩ s^−½^]	B [s^−½^]	R_ct_ [MΩ]	C_q_ [µF cm^−2^]
PET	4.879 ± .54	7.07 ± .14	.924 ±.085	178 ± 7.9	.565 ± 3.7e–4	74.91 ± 7.1	1.45 ± .06
SU-8	4.613 ± .39	7.41 ± 1.23	.868 ± .011	640 ± 13.6	.703 ± 3.4e–4	1.36 ± .13	3.72 ± .04

**TABLE II:** SUMMARY OF ACCELERATED AGING TEST FOR GRAPHENE MICROELECTRODE ARRAYS

Array	Final Status	Time Elapsed (days in 87 °C PBS)	Equivalent Lifetime (years in 37 °C)	Failure Mechanism
PET #1	Working	30	2.63	No failure
PET #2	Working	30	2.63	No failure
PET #3	Working	30	2.63	Minor PBS Permeation
PET #4	Working	30	2.63	No Failure
SU-8 #1	Failed	22	1.93	SU-8 cracks over electrode
SU-8 #2	Failed	17	1.49	SU-8 cracks over electrode
SU-8 #3	Failed	8	0.70	SU-8 cracks over wire
SU-8 #4	Failed	7	0.61	SU-8 cracks over wire
