# Supplementary material for: Evaluation of Durability of Transparent Graphene Electrodes Fabricated on Different Flexible Substrates for Chronic in vivo Experiments
Source: IEEE Trans Biomed Eng. Author manuscript; Available in PMC 2021 Nov 2. (PMC8560430; doi:10.1109/TBME.2020.2979475)
Supplement: supp1-2979475 [file NIHMS1639106-supplement-supp1-2979475.pdf]

Supplementary Information

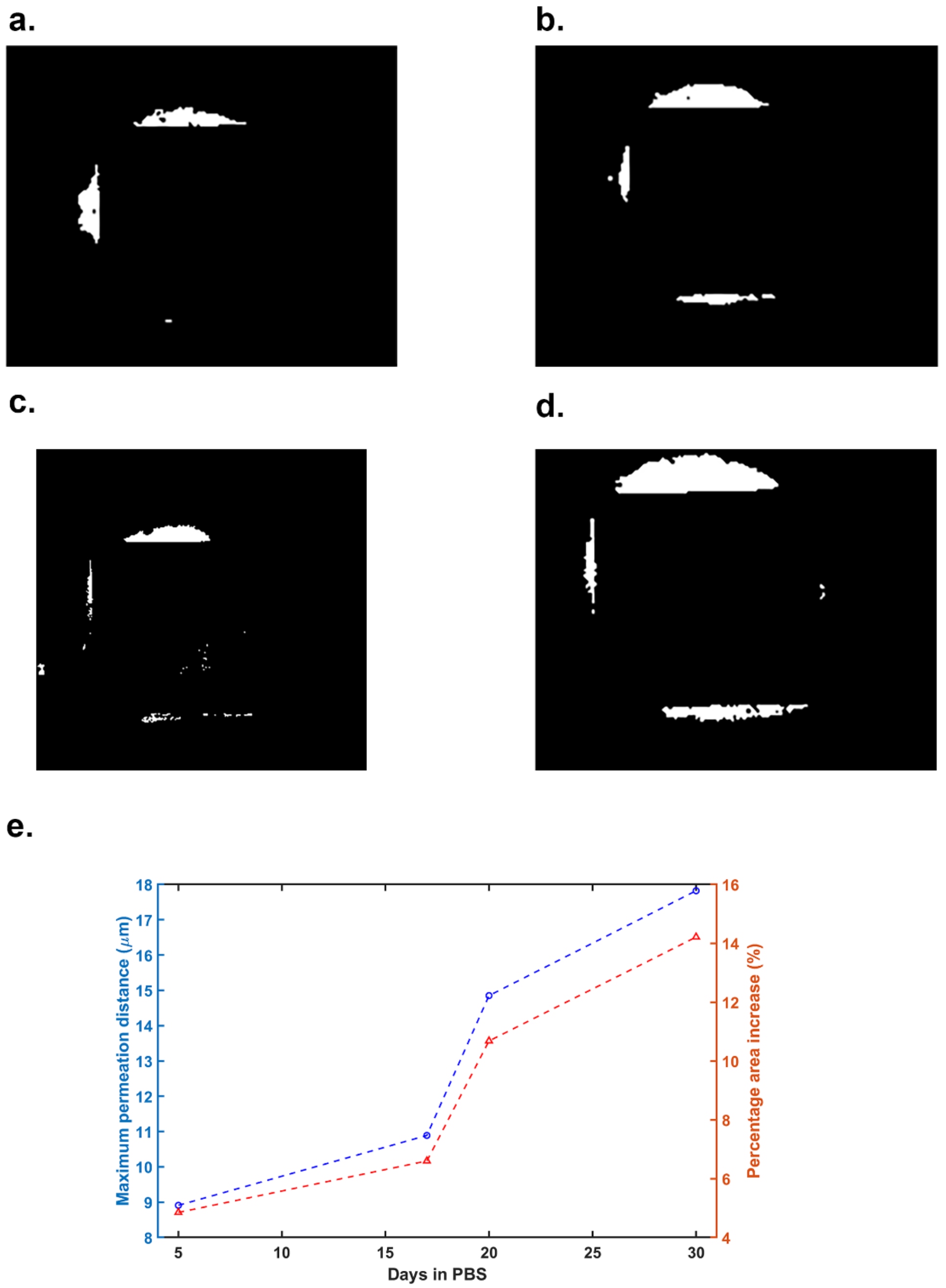

Supplementary Figure S1: Grey scaled images of microelectrode arrays fabricated on PET substrate over 30 days in solution, distinguishing PBS permeation around the electrode. (a) Day 5. (b) Day 17. (c) Day 20. (d) Day 30. (e) Maximum permeation distance and percentage area increase of the electrode as a function of days immersed in PBS solution.

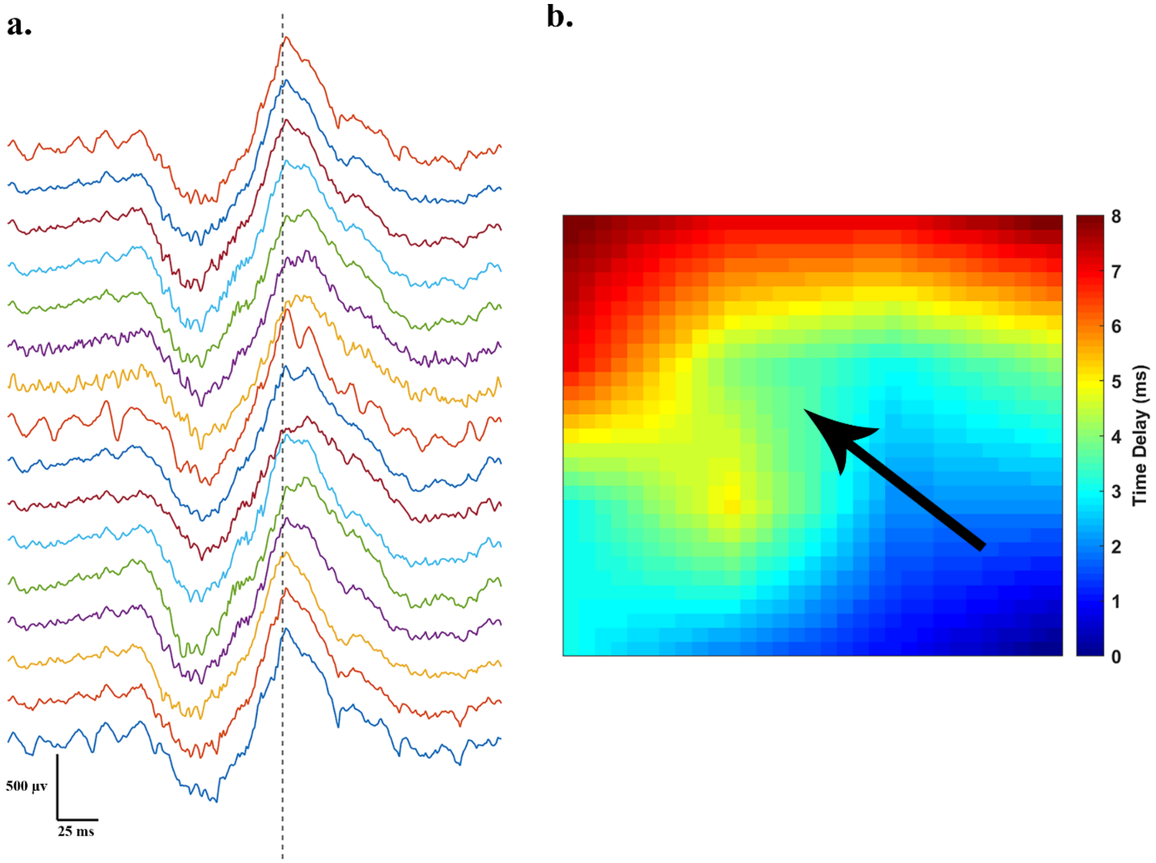

Supplementary Figure S2: (a) Representative multi-channel ECoG recordings demonstrating a time delay of spontaneous local-field potentials (LFPs) from different channels. (b) Time latency of the peak marked by the dashed line in panel (a). The black arrow indicates the direction of LFP propagation.

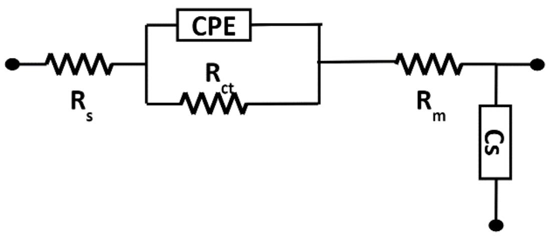

Supplementary Figure S3: R_s is the solution resistance. CPE is the constant phase element representing the double-layer capacitance, and R_{ct} represents the charge transfer resistance. R_m represents the resistance from the metal wires. C_s is the capacitance, which represents capacitive impedance across wires and channels [1].

Supplementary Table S1: Thermal and mechanical properties of soft polymer materials

Properties	Thermal Expansion Coefficient (ppm/°C)	Fracture Strain (%)	Young's Modulus (GPa)	Tensile Strength (Mpa)
**PET [2]**	**20-80**	**60-165**	**2.0-2.7**	**55**
**SU-8 2000 [3]**	**52**	**6.5**	**2.0**	**60**
**Parylene-C [4]**	**35**	**200**	**2.8**	**69**
**Polyimide [5]**	**52**	**19.1**	**2.5**	**231**

Supplementary Note 1. Equations for equivalent circuit models

Constant phase element:

$$Z_{CPE} = \frac{1}{(j\omega)^{A} C_{dl}}$$

Bounded Warburg element:

$$Z_{W_B} = \frac{W_B}{\sqrt{j\omega}} \tanh\left(\frac{\sqrt{j\omega}}{B}\right)$$

References:

[1] D. A. Robinson, "The electrical properties of metal microelectrodes," *Proceedings of the IEEE*, vol. 56, no. 6, pp. 1065-1071, 1968.

[2] "Polyethylene terephthalate Material Information." http://www.goodfellow.com/E/Polyethylene-terephthalate.html (accessed.

[3] "SU-8 Permanent Photoresists," ed.

[4] "Parylene Properties," ed.

[5] "Material Property Database," ed.
